# EMPATHY AND RELATIONSHIP QUALITY IN CHINESE PARENT–CHILD DYADS: THE MODERATING ROLE OF DAILY INTERACTION HOURS

**DOI:** 10.1093/geroni/igae098.2705

**Published:** 2024-12-31

**Authors:** Zewen Huang, Da Jiang

**Affiliations:** The Education University of Hong Kong, Hong Kong, China (People’s Republic); The Education University of Hong Kong, Hong Kong, China (People’s Republic)

## Abstract

Empathy has been consistently found to be associated with the quality of relationships in parents and children across the lifespan. However, little is known regarding whether and how such association would be influenced by the amount of time parents and children interact. We aimed to examine the relationship between empathy and relationship quality, and the moderating role in the relationship between aging parents and adult children from 99 parent-child pairs (Mage(parents)=48.3, SD=4.53, 79.8% women; Mage(children)=22.38, SD=3.49, 85.9% women) in this 14-day diary survey. We focused on both daily empathy collected in daily assessment and trait empathy collected in the pretest. By applying the actor-partner interdependence models and multilevel analysis, we found that the actor effects of daily and trait empathy on relationship quality were significant for both parents and children. Regarding the partner effects, parents’ daily empathy was significantly associated with children’s relationship quality, but not vice versa. Daily interaction hours moderated the associations. Specifically, for daily empathy, the impact of children’s daily empathy on parents’ relationship quality was significant only when parents and children interacted for a longer (vs. shorter) period of time. For trait empathy, the relationship between parents’ trait empathy and their own relationship quality, and that between parents’ trait empathy and their children’s relationship quality were stronger among parent-child dyads reporting shorter (vs. longer) average daily interaction hours over the 14 days. These findings elucidated the complex associations between empathy and relationship quality in parent-child relationships by highlighting the moderating role of daily interaction hours.

